# Purification of aqueous orange II solution through adsorption and visible-light-induced photodegradation using ZnO-modified g-C_3_N_4_ composites†

**DOI:** 10.1039/d4ra01481b

**Published:** 2024-06-04

**Authors:** Mahmudul Hassan Suhag, Aklima Khatun, Ikki Tateishi, Mai Furukawa, Hideyuki Katsumata, Satoshi Kaneco

**Affiliations:** a Department of Applied Chemistry, Graduate School of Engineering, Mie University Tsu Mie 514-8507 Japan hidek@chem.mie-u.ac.jp; b Department of Chemistry, University of Barishal Barishal 8254 Bangladesh; c Mie Global Environment Center for Education & Research, Mie University Tsu Mie 514-8507 Japan

## Abstract

Semiconductor-based remediation enables environmentally friendly methods of removing aqueous pollutants. Simply fabricated ZnO modified g-C_3_N_4_ composites were utilized as bifunctional adsorptive photocatalysts for orange II removal from aqueous solution through adsorption and photocatalysis processes. The adsorption isotherm data of the g-C_3_N_4_ (g-CN) and ZnO modified g-C_3_N_4_ (ZCN) composites on orange II solution were better fitted with the Langmuir isotherm compared to the Freundlich isotherm. The maximum adsorption capacity for ZCN-2.5 was slightly higher than that of bare g-CN. According to the adsorption thermodynamics investigation of ZCN-2.5 in orange II solution, the positive values of Gibb's free energy change (Δ*G*^0^) suggested a non-spontaneous adsorption process. Furthermore, the negative values of entropy change (Δ*S*) and enthalpy change (Δ*H*) indicated the decrement of randomness and exothermic nature during the adsorption process, respectively. The photocatalytic degradation kinetics of g-CN and ZCN composites indicated that the degradation process follows the pseudo-first-order reaction kinetic. The degradation rate of orange II with the ZCN-2.5 composite was 6.67 times higher than that obtained with bare g-CN. Possible adsorption and photocatalytic mechanisms have been proposed.

## Abstract 2

Purification of aqueous orange II solution using ZnO-modified g-C_3_N_4_ composites as bifunctional adsorptive photocatalysts.

## Graphical abstract

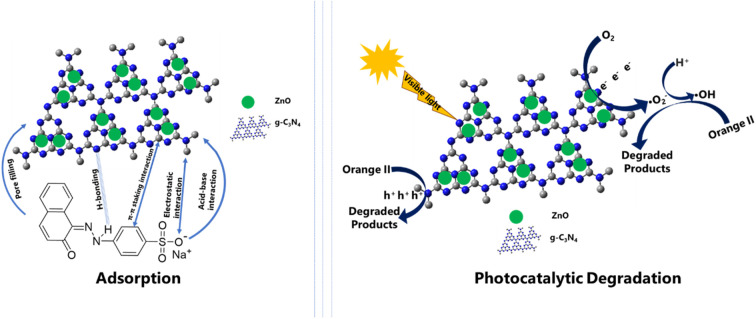

## 1. Introduction

Dyes are basically organic substances that are usually embedded in fabrics or surfaces for coloring or pigmentation, and most of the dyes are typically large molecules.^1^ Several industries, including textile, paper, rubber, leather tanning, plastic, food processing, cosmetics, printing, and dye manufacturing industries, extensively use synthetic dyes.^1–6^ In order to measure the surface area of activated sludge, dirt and effluent purification, and the tracing of groundwater, synthetic dyes are also used.^1^ The aquatic ecosystem is adversely affected by the improper release of highly colored effluent containing dyes from these industries, which also intensifies ecotoxicological effects, reduces light penetration and photosynthesis, and increases the risk of cancer and mutagenicity in humans. Consequently, effluent from industrial dye discharge has become a major threat to the environment.^5–7^ Therefore, industrial effluents that contain dyes should be properly treated before discharge into the environment. Multiple techniques have been employed to remove dyes from effluent, including adsorption,^8,9^ membrane separation,^10^ electrocoagulation,^11^ electrochemical,^12,13^ biological,^14,15^ and advanced oxidation processes.^16–18^ Among these techniques, adsorption is regarded as one of the most convenient and efficient methods of removing dyes due to its high efficiency, ease of use, and availability of adsorbent materials.^1,19^ In view of their large surface area and adsorption efficacy, activated charcoal, fly ash, silica gel, molecular organic framework, and other conventional adsorbent materials have been used in the dye contaminated wastewater adsorption studies. However, the use of these adsorbents is costly and hostile to the environment.^20^

In addition, oxidative degradation known as photocatalytic degradation of dye-containing effluents under solar irradiation has drawn a lot of interest because of its cost-effectiveness, cleanliness, high efficacy, and long-term sustainability. For instance, it is essential to pick, plan, and develop materials that have both adsorption and photocatalytic activity for the purpose of removing dye from effluent. The bifunctional adsorptive photocatalysts are required to have high capacity, outstanding compatibility, and prolonged recyclability.^21–23^

The textile dye orange II (4-(2-hydroxy-1-naphthyl)azobenzene) is a monoazo, anionic, and acidic dye. It is extensively utilized in the industrial sector because of its durability. Due to its multi-step elimination process and ability to infect people through the food chain, it poses a threat to the environment. Due to its lower biodegradability, wastewater treatment plants can't degrade it perfectly. The excessive use of orange II dye will significantly pollute water bodies, and prolonged exposure to this dye can result in a number of health problems for humans.^24–26^ Many research works have been reported on the adsorption^27–33^ and photocatalytic degradation^24–26^ of orange II dye using different types of adsorbents and photocatalysts, respectively.

Graphitic carbon nitride (g-C_3_N_4_, abbreviated as g-CN), a carbon-based material, has attracted a lot of interest and is used in a variety of applications, including photocatalysis,^34,35^ electrogenerated chemiluminescence,^36^ lithium-ion batteries,^37^ hydrogen generation,^38^ fluorescent sensors,^39^ and adsorption.^22^ The hexagonal ring-based C–N network of g-CN is composed of tri-*s*-triazine units. Due to the presence of delocalized π-electrons in tri-*s*-triazine units, aromatic compounds are attracted to g-CN *via* π–π interaction and hydrophobic effect. Thus, g-CN emerges as a potential efficient adsorbent for the adsorption of dyes with aromatic structures. However, the efficacy of g-CN as an adsorbent for dye adsorption is inadequate.^40^ Therefore, it should be necessary to enhance the adsorption capacity of g-CN by increasing the surface area and pore size, changing the π–π stacking, forming an induced electric field, and increasing the H-bond ability.^19,21,22,40^ Several modifications of the g-CN structure have been reported to enhance its adsorption ability. For instance, Yousefi *et al.* prepared oxidized g-CN as an effective adsorbent for organic dyes and tetracycline for water remediation.^19^ Ren *et al.* reported the preparation of carbon-doped g-CN for the adsorption of methylene blue from an aqueous solution. The adsorption kinetics, isotherm, and thermodynamics were also studied.^40^ Yan *et al.* synthesized sodium-doped g-CN for the removal of aqueous contaminants *via* adsorption and photocatalysis. It was reported that the sodium-doped g-CN is a potential adsorbent for the adsorption of methylene blue, acriflavine, azure blue, rhodamine B, safranin O, and methyl orange dyes.^22^ Panneri *et al.* reported carbon-doped g-CN as an effective adsorptive photocatalyst for the removal of tetracycline antibiotics from waste water.^21^ Khosrowshahi and Razmi studied the application of sunflower stalk g-CN nanosheets as a green adsorbent in the extraction of polycyclic aromatic hydrocarbons from the liquid g-CN nanosheets phase.^41^ Wang *et al.* reported the application of α-Fe_2_O_3_/g-CN composites for the synergistic adsorption and photodegradation of methyl orange and methylene blue dyes.^42^

Some inherent characteristics of g-CN, especially low charge separation, poor quantum efficiency, and quick recombination rate of the photogenerated e^−^–h^+^ pairs, decrease its visible-light-responsive photocatalytic efficiency.^43,44^ To boost the photocatalytic activity of g-CN, some attempts have been focused such as by elemental doping,^45–47^ molecular doping,^48^ and coupling with other semiconductors, including metal oxides,^49–51^ metal sulfides,^52,53^ and others.^54,55^ It was reported that ZCN composites showed excellent photocatalytic activity due to the appropriate band gap alignment of g-C_3_N_4_ and ZnO. For example, Paul *et al.* fabricated the ZnO/g-C_3_N_4_ composites for enhanced photocatalytic degradation of methylene blue solution with visible light irradiation.^56^ Zhang *et al.* synthesized the ZnO/g-C_3_N_4_ composites, which were applied to the efficient photocatalytic degradation of methyl orange and tetracycline under the irradiation of visible light.^57^ Ismael *et al.* prepared the ZnO/g-C_3_N_4_ composites, and the effective photocatalytic activity of the prepared composite was evaluated under visible light irradiation.^43^ Gayathri *et al.* reported the improved photocatalytic degradation of methylene blue and acid blue 113 dyes with the irradiation of sunlight.^50^ In our previous studies, we reported the different ZnO/g-C_3_N_4_ composites for the enhanced photocatalytic degradation of endocrine-disrupting chemical bisphenol *E* and reduction of hexavalent chromium solution under visible light irradiation.^34,58^

In this paper, the adsorption and photocatalytic activity for orange II dye removal from aqueous solution using ZCN composites were reported as bifunctional adsorptive photocatalysts. The adsorption isotherms and thermodynamics properties were also studied. The photocatalytic degradation kinetics of the composites were also testified. The photocatalytic degradation efficiency of the composites was compared with their adsorption efficacy.

## 2. Materials and methods

### 2.1. Fabrication of the ZCN composites

The ZCN composites were fabricated according to our published paper.^34,58^ In brief, ZCN composites were synthesized by the single-step thermal calcination of homogeneous mixtures of zinc acetate dihydrate and urea with different ratios. Typically, different amounts of zinc acetate dihydrate and 20 g of urea were homogeneously mixed with a dispersion of 5 mL of water in an alumina crucible. Then it was covered by a lid and aluminum foil and calcined at 550 °C for 2 hours with a heating increasing rate of 2 °C min^−1^ using an electric muffle furnace. Lastly, the calcined samples were ground manually into different colored powders. The obtained composites were labeled as ZCN-1, ZCN-2.5, ZCN-5, ZCN-10, and ZCN-15, in accordance with the various amounts of zinc acetate dihydrate that were used 0.2, 0.5, 1.0, 2.0, and 3.0 g, respectively. The content of the ZnO in the composites has been determined by thermal gravimetric analysis using a muffle furnace. The estimated ZnO contents are 7.2, 20.4, 30.0, 39.1, and 45.3% in the ZCN-1, ZCN-2.5, ZCN-5, ZCN-10, and ZCN-15 composites, respectively. Urea and zinc acetate dehydrate were calcinated at similar temperatures separately to prepare bare g-CN and ZnO, respectively.

### 2.2. Characterization

The FTIR spectra of the ZCN composites and bare g-CN were recorded on a PerkinElmer spectrometer (SPECTRUM 100 FTIR) at 5 number of scans with an attenuated total reflection assemblage and a resolution of 0.5 cm^−1^. XRD patterns of the prepared ZCN composites, ZnO, and g-CN were analyzed by utilizing a Rigaku RINT Ultima-IV diffractometer with Cu K_α_ radiation in a scan range of 10–80° at a scan rate of 0.04° s^−1^. The XPS of ZCN-2.5 composite and g-CN were characterized by using a PHI Quantera SXM photoelectron spectrometer with Al K_α_ radiation. The C 1s peak at 284.8 eV was used as a reference to adjust the binding energies. In order to observe the surface morphology, SEM images of the all prepared ZCN composites, ZnO, and g-CN and EDS elemental mapping of the ZCN-2.5 composite were recorded using a JEOL JEM-1400 Flash SEM. The TEM image of the ZCN-2.5 composite (slurry on methanol) was analyzed on a JEOL JEM-1011 TEM at 100 kV. In order to attain BET surface area, average pore size and total pore volume of all ZCN composites and g-CN, N_2_ adsorption–desorption isotherms were recorded on the BELSORP-miniII (MicrotracBEL) apparatus. The UV-vis DRS of all composites, ZnO and g-CN were inspected by a JASCO V-750 UV-vis instrument equipped with an integrating sphere adaptor. Photoluminescence (PL) spectra of all composites, ZnO and g-CN were attained by a Shimadzu fluorescence spectrophotometer (RF-5300PC) with an excitation wavelength of 340 nm. The EIS measurement of all composites and g-CN were analyzed on an electrochemical Versa STAT 3 workstation (Princeton Applied Research) equipped with a conventional three-electrode system. In here, uniform slurry of the sample by Nafion solution was coated on a fluorine-doped tin oxide glass plate to make the working electrode, and an aqueous solution of Na_2_SO_4_ (0.5 mol L^−1^) was utilized as the electrolyte.

### 2.3. Adsorption study

The orange II aqueous solution (100 mg L^−1^) was prepared as the stock solution in a volumetric flask. Also, different concentrated diluted solutions were prepared by proper dilution. To study the adsorption of orange II solution by g-CN and different ZCN composites, a series of batch adsorption analyses were conducted. The relative standard deviation (RSD) of each experiment was calculated with triplicate measurements, and the observed RSD values were less than 10%. A Pyrex glass cell (50 mL) was used for the adsorption experiment. Herein, 30 mg of adsorbents and 30 mL of orange II solution were poured into the glass cell, covered by aluminum foil, and magnetically stirred for the adsorption test. Throughout the experiment, 1.5 mL of sample solution was withdrawn at different time intervals. Then the collected sample solution was centrifuged for 5 min at 12 000 rpm to isolate the adsorbents. The absorbance of the supernatant was measured using a UV-vis spectrophotometer at 485 nm wavelength to assess the concentration of orange II solution.

### 2.4. Photocatalytic degradation study

Thirty milliliters of orange II solution (10 mg L^−1^) and 30 mg of photocatalyst of were put into 50 mL of a Pyrex glass cell. The adsorption process was then let run in the dark for 30 minutes to obtain adsorption–desorption equilibrium. Later, the solution was illuminated by using a light-emitting diode (LED) lamp (OptoCode LDA14L-G/100W) with a UV (400 nm) cutoff filter (Y-44, HOYA). The light source was set up on one side of the reaction cell. Through the degradation, 1.5 mL of the solution was collected every 30 minutes and centrifuged at 12 000 rpm for 5 minutes to separate the photocatalyst from the liquid phase. Then the concentration of the orange II dye liquid phase was evaluated by measuring the absorbance with a UV-vis spectrophotometer at 485 nm wavelength. The reproducibility of the photocatalytic degradations have also been investigated, and relative standard deviations were observed within 10% for more than three runs.

## 3. Results and discussion

### 3.1. XRD analysis

**Fig. 1:**
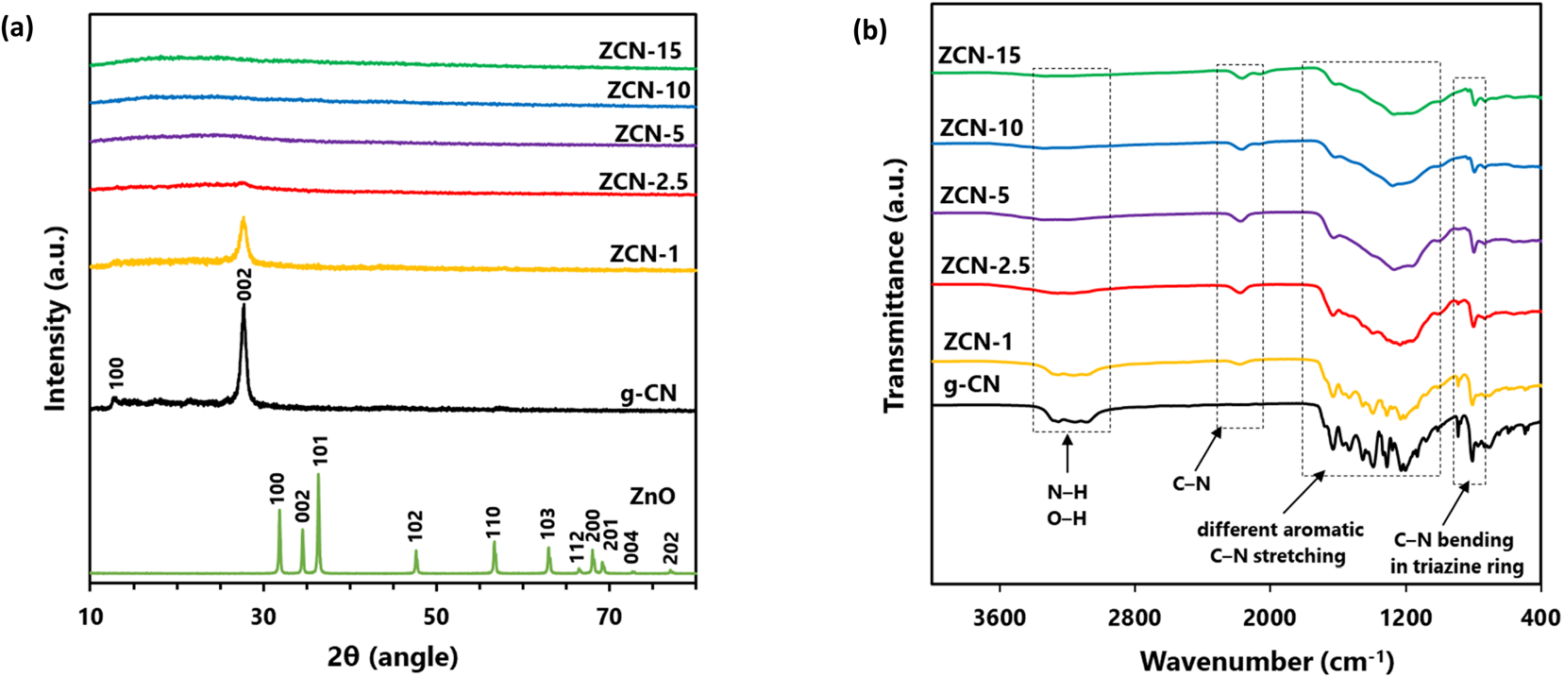
(a) XRD patterns of ZnO, g-CN and different ZCN composites and (b) FTIR spectra of g-CN and all fabricated ZCN composites.

In order to assess the crystalline nature of the prepared photocatalysts, XRD analysis was performed. Fig. 1a shows the powder XRD patterns of prepared g-CN, ZnO, and a series of fabricated ZCN composites. Two distinct peaks were observed in the XRD pattern of g-CN at 12.82 and 27.66° 2*θ* values, which can be indexed as (100) and (002) planes, respectively. The observed small peak at 12.82° was related to the periodic reflection of the *s*-triazine units. Moreover, the observed intense peak at 27.66° was associated with the long-range interlayer conjugated aromatic C–N stacking.^44,59^ Several distinct diffraction peaks were found in the XRD pattern of prepared ZnO at 31.94, 34.64, 36.32, 47.68, 56.72, 63.01, 66.68, 68.06, 69.12, 73.12, and 77.52° 2*θ* values, which could resemble the plane of (100), (002), (101), (102), (110), (103), (200), (112), (201), (004), and (202), respectively, of the wurtzite structure of hexagonal ZnO.^60^ A peak detected at about 27° 2*θ* value in the XRD pattern of ZCN-1 and ZCN-2.5 represents the diffraction phase of the g-C_3_N_4_. The peak in the XRD pattern of ZCN-2.5 was weaker and broader than that of ZCN-1, which corresponded to the decrease of the g-CN crystal phase. Moreover, the peak was boarded and disappeared in the XRD patterns of ZCN-5, ZCN-10, and ZCN-15, indicating the loss of g-CN crystallinity in the composites (Fig. 1a and S1†). Interestingly, no ZnO-related diffraction peak was observed in the XRD patterns of all ZCN composites, which denotes that the crystallinity of the ZnO was lost during the formation of all composites.^56^ Hence, the fabricated ZCN composites containing higher content of ZnO exist in amorphous state.

### 3.2. FTIR analysis

To study the bonding of ZnO with g-CN in the fabricated ZCN composites, the g-CN and all ZCN composites were characterized by FTIR spectroscopy (Fig. 1b). In the FTIR spectra of pure g-CN, an intense peak was observed at 807 cm^−1^, which is attributed to the bending mode of the heterocyclic C–N bond in the *s*-triazine ring.^61^ The peak was shifting to lower wave number and decreasing intensity with increasing the ZnO content in the ZCN composites, which suggested the bond strength of heterocyclic C–N was decreased by the interaction of ZnO and g-CN. Several peaks were observed at 1200 to 1650 cm^−1^in the FTIR spectra of g-CN, corresponding to the stretching modes of C–N and C=N bonds in the aromatic heterocycles of the g-CN framework.^62^ The intensity of the peaks decreased with increasing ZnO content in the ZCN composites and merged for higher ZnO-containing composites. A broad peak with small intensity was observed at 3000 to 3450 cm^−1^ associated with the stretching modes of O–H bonds in adsorbed moisture and water and uncondensed N–H bonds.^61,62^ However, the peak was weaker or disappeared for the ZnO-incorporated composites. In addition, a new peak with lower intensity appeared at about 2160 to 2180 cm^−1^ in the FTIR spectra of all ZCN composites, which is absent in the FTIR spectra of g-CN. The new peak indicated the formation of new C–N bond along with sp^2^ C–N bonds in the g-CN network.^56^ The results supported the chemical interaction between ZnO and g-CN during the formation of ZCN composites.

### 3.3. XPS analysis

**Fig. 2:**
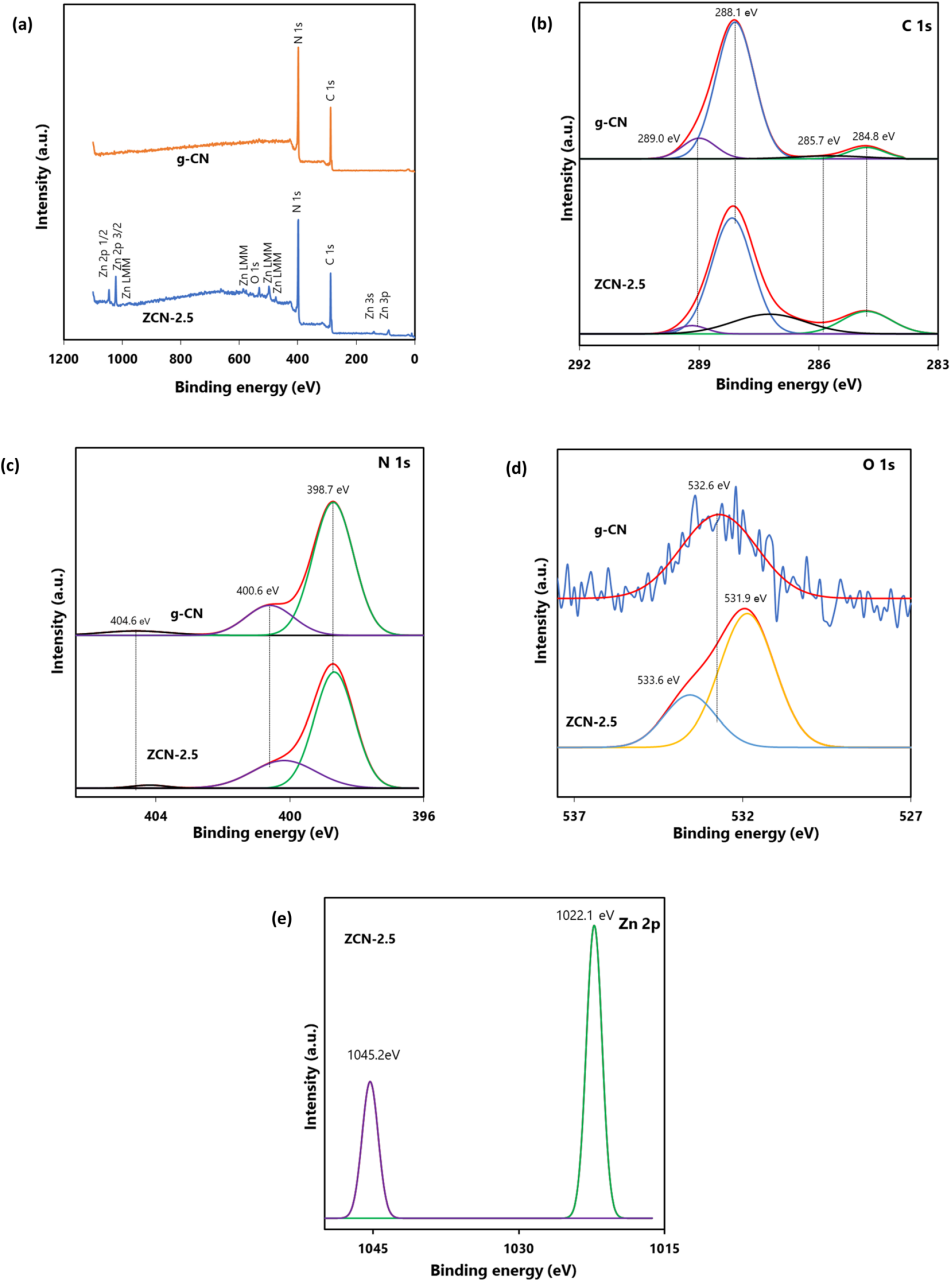
(a) Survey XPS spectra of g-CN and ZCN-2.5; overlap high resolution XPS (b) C 1s, (c) N 1s and (d) O 1s spectra of g-CN and ZCN-2.5 and (e) High resolution XPS Zn 2p spectra of ZCN-2.5.

To specify the sample element state, XPS analysis was employed (Fig. 2). The full scan survey XPS spectra of ZCN-2.5 composite and g-CN demonstrated that g-CN is composed of C and N elements, whereas the ZCN-2.5 composite is composed of Zn, O, C, and N components (Fig. 2a and Table S1†). The findings indicated that the ZCN-2.5 composite was pure and well-formed.^50,63^ The high-resolution spectra of C 1s, N 1s, O 1s, and Zn 2p are depicted in Fig. 2b–e. As illustrated in Fig. 2b, four peaks are fitted in the high resolution XPS spectra of C 1s for ZCN-2.5 composite and g-CN. For bare g-CN, the binding energies of these four peaks were about 284.8, 285.7, 288.1, and 289.0 eV, which are attributed to the C=C or C–C bond, sp^3^ C–N bond, N=C–N groups in the *s*-triazine ring, and the unavoidable oxidized carbon C=O bond, respectively.^64^ The high resolution XPS spectra of N 1s for both g-CN and ZCN-2.5 are associated with three distinct peaks (Fig. 2c). In the case of bare g-CN, the peak located at 398.7 eV is related to sp^2^ nitrogen in the triazine ring (N=C–N). The peaks positioned at 400.6 eV is regarded as the bridging nitrogen N atoms (N–(C)_3_) and terminal amino groups (C–NH_2_), and at 404.6 eV is regarded as the charging effect in heterocycles or triazine.^40,59^ In comparison to g-CN, ZCN-2.5 showed a decrease in peak intensity of the C 1s and N 1s XPS spectra, attributed to the N=C–N group. This result suggested that the ZnO coupling restacks the π–π conjugation in the aromatic ring structure of g-CN.^64^ Furthermore, the major peak at higher resolution C 1s of ZCN-2.5 is shifted to higher binding energies than that of g-CN, indicating that the electrons are moved from g-CN to ZnO. This provides strong evidence that electrons are transferred from g-CN to ZnO in the ZCN-2.5 heterojunction, resulting in the formation of an induced electric field.^65,66^ As indicated in Fig. 2d, the O 1s high resolution XPS spectrum of g-CN showed only one peak at 532.6 eV that was caused by surface absorbed H_2_O or O–H groups.^44^ In contrast, there were two peaks in the ZCN-2.5 composite O 1s high resolution XPS spectrum, which were situated at 531.9 and 533.6 eV, respectively. The major peak (531.9 eV) is assigned to the contribution of O_2_^2−^ ions in the wurtzite structure of ZnO, while the tiny peak (533.6 eV) is associated with –OH groups or the H–O–H bond of adsorbed H_2_O on the composite surface.^67^ In addition, the peak of Zn 2p_3/2_ is at 1022.1 eV, and Zn 2p_1/2_ is at 1045.2 eV indicated the existence of Zn^2+^ in the ZCN-2.5 composite (Fig. 2e).^50^ The binding energy difference between two peaks was 23.1, which is consistent with the standard ZnO reference value.^68,69^ Also, Zn–N bonding can be detected by the Zn LMM Augur peaks in the ZCN-2.5 composite survey spectrum.^64^

### 3.4. Morphological analysis

The morphology of prepared pure ZnO, pure g-CN, and all ZCN composites was investigated by SEM analysis (Fig. S2†). The SEM image of ZnO shows spherical particles with different sizes.^59^ The sheet-like layered structure was revealed in the SEM image of g-CN.^70^ The SEM image of the ZCN-1 composite shows the existence of a sheet-like layered structure of g-CN with closely arranged ZnO particles. However, the surface morphology of other fabricated ZCN composites is blurred, which indicates the g-CN and ZnO are collapsed and aggregated during the composite formation.

**Fig. 3:**
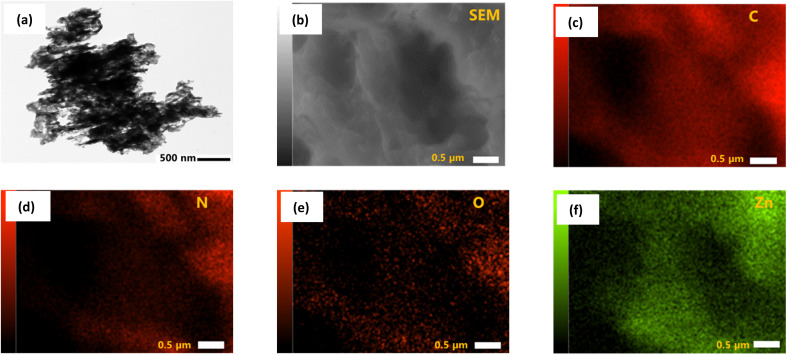
(a) TEM image and (b–f) EDS elemental mapping with corresponding SEM image of ZCN-2.5.

The existence of spherical ZnO in the g-CN sheet is displayed in the TEM image of the ZCN-2.5 composite (Fig. 3a). Further, in order to demonstrate the presence of C, N, O, and Zn in the ZCN-2.5 composite, EDS elemental mapping was analyzed. It is apparent in Fig. 3b–f that C, N, O, and Zn are uniformly distributed on the composite's surface.

### 3.5. Textural characterization

The textural properties such as surface area, average pore volume, and pore diameter of the pure g-CN and all fabricated ZCN composites were analyzed by measuring the physical adsorption ability of nitrogen. The N_2_ adsorption desorption isotherms with pore size distribution curves of pure g-CN and ZCN composites are shown in Fig. S3.† It was confirmed that the isotherms are attributed to the classical IV type with the H3 hysteresis loop, suggesting the existence of mesopores and slit-like pores within the structure.^71,72^ From Table 1, it can be seen that the Brunauer–Emmett–Teller (BET) surface area of ZCN-1 was increased to a low extent, and ZCN-2.5 was slightly decreased compared to pure g-CN. By further increasing the ZnO content in the composite, the BET surface area was drastically decreased. The total pore volume and average pore diameter of ZCN-1 and ZCN-2.5 increased compared to pure g-CN. Furthermore, in the case of higher ZnO containing composites, these are decreased compared to pure g-CN. Excess ZnO in the composite may cause aggregation and block the original mesopores and slit-like pores in the g-CN segment.^71,72^

**Table 1:** Surface area, pore volume, and pore diameter of the pure g-CN and all fabricated ZCN composites

Materials	BET surface area (m^2^ g^−1^)	Total pore volume (cm^3^ g^−1^)	Average pore diameter (nm)
g-CN	55.67	0.44	31.88
ZCN-1	56.00	0.53	37.64
ZCN-2.5	49.28	0.43	35.57
ZCN-5	21.4	0.08	14.9
ZCN-10	10.4	0.08	19.5
ZCN-15	7.49	0.06	30.56

### 3.6. UV-vis DRS analysis

**Fig. 4:**
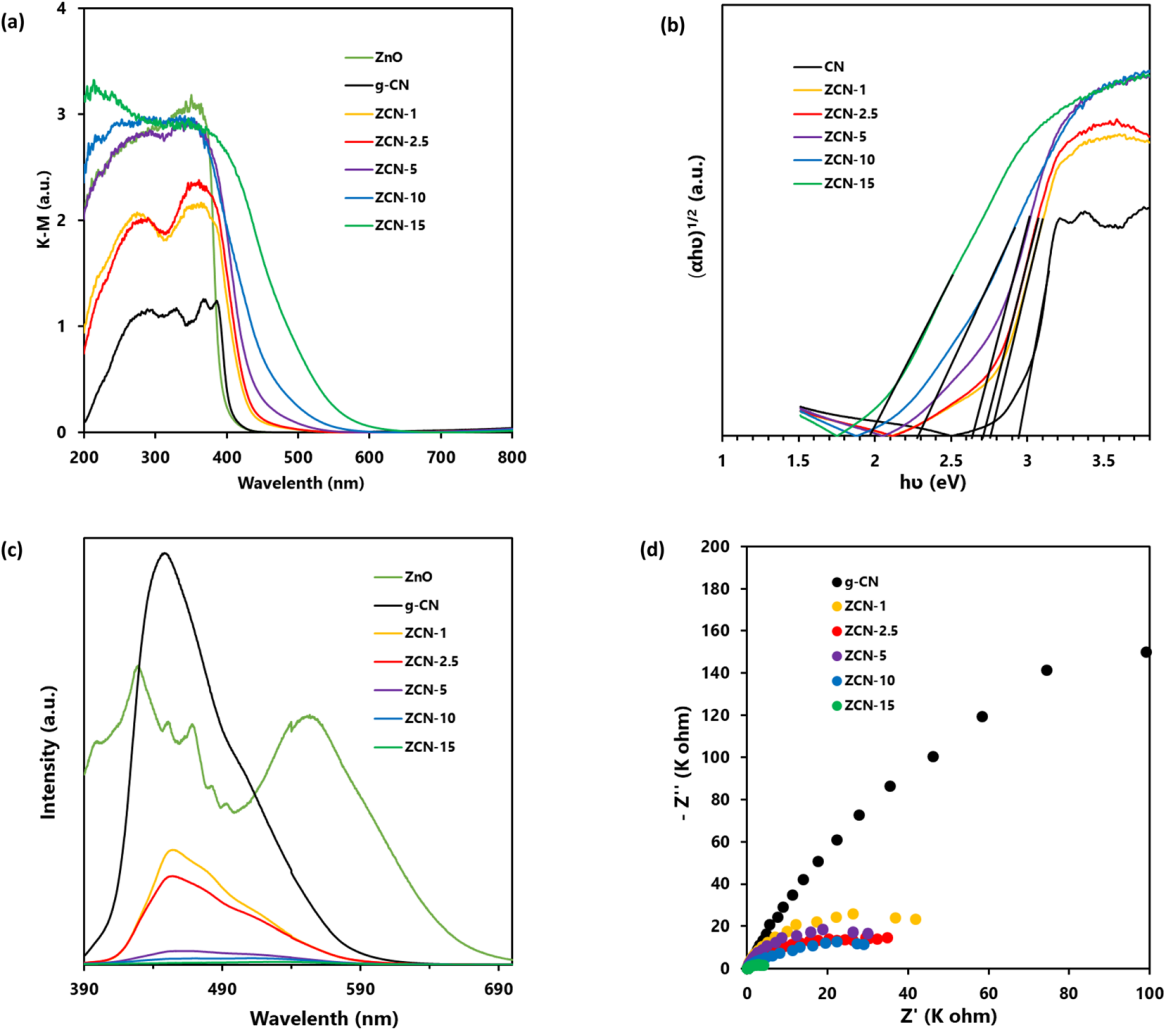
(a) Kubelka–Munk function of UV-vis DRS and (c) PL spectra (upon the excitation at 340 nm wavelength) of ZnO, g-CN, and all fabricated ZCN composites. (b) Tauc plot and (d) EIS resultant Nyquist plot of g-CN and different ZCN composites.

In order to investigate the light absorption ability of prepared g-CN, ZnO, and all ZCN composites, the UV-vis DRS was conducted at room temperature. Fig. 4a shows the Kubelka–Munk-transformed UV-vis DRS profile of prepared ZnO, g-CN, and all ZCN composites. It is clearly seen that the absorption edge of ZnO is at about 390 nm, which indicates that it is non-responsive to visible light.^73^ The absorption edge of g-CN was seen at about 430 nm, which is responsive to visible light.^43^ Moreover, the absorption edges of the ZCN composites were red shifted to higher wavelength compared to pure ZnO and g-CN, with increasing the ZnO content in the composite. The enhancement of the visible light absorption ability of the composites indicates the interaction between ZnO and g-CN through chemical bonding.^43^ Furthermore, the band gap of the samples was calculated using eqn (1).

1 *αhv* = *A*(*hv* − *E*_g_)^*n*^

where *α* is the absorption coefficient, *hν* is the photoenergy, *A* is a constant, and *E*_g_ is the optical band gap. The value of *n* is 2 or ½ for indirect transition (*e.g.*, g-CN and ZCN composites) or direct (*e.g.*, ZnO) transition, respectively.^57^ As shown in Fig. 4b and S4a,† the estimated band gap values of ZnO, g-CN, ZCN-1, ZCN-2.5, ZCN-5, ZCN-10, and ZCN-15 are 3.20, 2.93, 2.74, 2.70, 2.62, 2.28, and 1.96, respectively. As shown in Fig. S4b,† the absorption edge and band gap change of the composites represent their color change.

### 3.7. PL analysis

The recombination of charge carriers in the prepared pure g-CN, ZnO, and all fabricated ZCN composites was evaluated using PL analysis (Fig. 4c). In general, the frequency of recombination of electron and hole pairs is closely proportional to the intensity of the PL spectra.^57^ Upon excitation at 340 nm, the g-CN showed a very intense peak at wavelength of 440 nm. Furthermore, the ZnO showed several intense peaks. The findings suggested the higher electron hole pairs recombination in g-CN and ZnO separately. On the other hand, the PL intensity of the ZCN composites was drastically decreased and slightly shifted compared to the peak of g-CN with the increasing ZnO content in the composite, suggesting the decrement of electron hole pairs recombination. It may be caused by increments in electron transfer from the g-CN to ZnO upon the increase in ZnO content in the composite.^74^

### 3.8. EIS analysis

To learn about the process of photoinduced charge migration and transfer, EIS measurements were carried out. In the EIS resultant Nyquist plot, a smaller arc radius generally denotes reduced charge transfer resistance and more charge separation.^43^ As shown in Fig. 4d, the composites had smaller curvature radius compared to pure g-CN, which suggested the enhanced photoinduced charge separation efficiency of g-CN after the formation of composites.

### 3.9. Adsorption of orange II solution

To study adsorption of Orange II solution (10 mg L^−1^), 30 mg of adsorbents was used at different time intervals for adsorption capacity (*q*_*t*_) of g-CN and ZCN composites. The experiments were carried out at room temperature and the pH was 3.0. Eqn (2) was used for the quantity of *q*_*t*_ (mg g^−1^) of various adsorbents at time *t*.^40^2
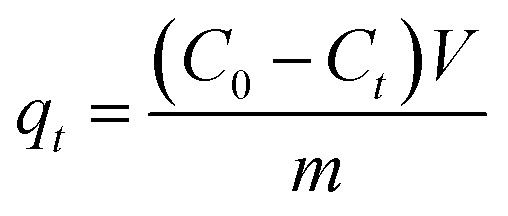
where *m* is the adsorbent mass and *V*, *C*_0_, and *C*_*t*_ are the volume, initial concentration, and concentrations at time *t* of the dye solution, respectively.

**Fig. 5:**
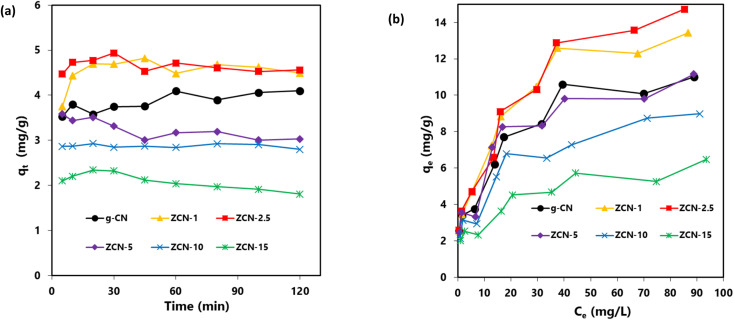
(a) Effect of contact time on the adsorption of orange II by prepared pure g-CN and different ZCN composites; Orange II solution, 10 mg L^−1^ (30 mL); adsorbents, 30 mg. (b) Effect of equilibrium concentration on the adsorption of orange II prepared pure g-CN and different ZCN composites; Orange II solution, 30 mL (3–100 mg L^−1^); adsorbents, 30 mg.

As depicted in Fig. 5a, the equilibrium between adsorption and desorption was almost fully accomplished in about 30 minutes. It was also observed that the quantity of adsorption capability in ZCN composites enhanced with increasing amount of ZnO from bare g-CN to ZCN-2.5 composite and further decreased with increasing ZnO quantity.

For different dye adsorbents with various initial concentrations (3–100 mg L^−1^), the quantity of adsorbed adsorbates *q*_e_ (mg g^−1^) at equilibrium condition (30 minutes) was evaluated by eqn (3).^75^3
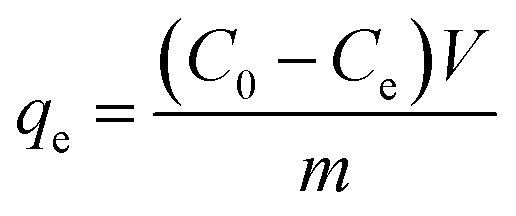
where *m* is the adsorbent mass and *V*, *C*_0_, and *C*_e_ are the volume, initial concentration, and equilibrium concentrations at time *t* of the dye solution, respectively. As shown in Fig. 5b, it was observed that the *q*_e_ values increased with the *C*_e_ value of adsorbed dye solution.

Generally, a number of equilibrium adsorption isotherm equations are used to describe experimental adsorption data. To understand the surface properties, the highest adsorption capacities and adsorbate affinity to adsorbents can be predicted using several adsorption isotherms. Hence, it is important to know the adsorption isotherm that fits the data acquired the best. Two widely utilized mathematical models are the Freundlich isotherm and the Langmuir isotherm models. The Langmuir model assumes a monolayer coverage and uniformity of all adsorbent sorption sites. As seen in eqn (4), the Langmuir model is as follows:^22^4
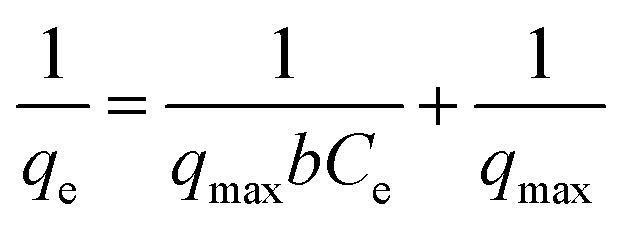
where *q*_max_ represents the maximum adsorption capacity and *b* is the Langmuir adsorption constant.

**Fig. 6:**
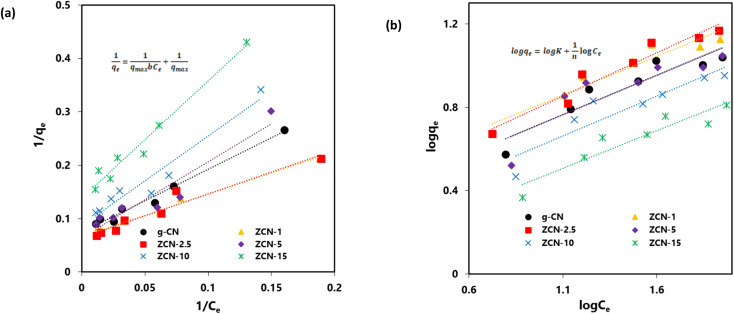
(a) Langmuir isotherm and (b) Freundlich isotherm for adsorption of Orange II dye by pure g-CN and different ZCN composites.

The data fittings of the Langmuir isotherms for the adsorption of orange dye on the composites are shown in Fig. 6a, and the maximum adsorption capacity (*q*_max_) and Langmuir adsorption constant (*b*) are presented in Table 2.

**Table 2:** Langmuir isotherm model fitting results

Adsorbents	Intercept	Slope	*q*_max_	*b*	*R* ^2^
g-CN	0.074	1.778	13.441	0.042	0.983
ZCN-1	0.067	0.792	15.015	0.084	0.987
ZCN-2.5	0.064	0.821	15.528	0.078	0.987
ZCN-5	0.065	1.420	15.480	0.045	0.903
ZCN-10	0.087	1.679	11.507	0.052	0.942
ZCN-15	0.138	2.211	7.246	0.062	0.970

In addition, the Freundlich isotherm model implies that all adsorption sites are heterogeneous and that the coverage is multilayer. As seen in eqn (5), the Freundlich model is as follows:^22^5
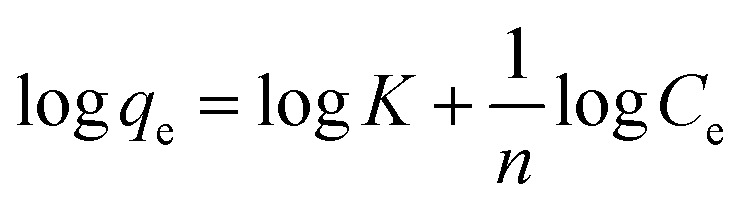
where *K* is the Freundlich adsorption constant and *n* represents the intensity of the adsorption.

The data fittings of the Freundlich isotherms for the adsorption of orange dye on the composites are shown in Fig. 6b, and the Freundlich adsorption constant (*K*) and adsorption intensity (*n*) are presented in Table 3. Based on the *R*^2^ values in Tables 2 and 3, the Langmuir model isotherm represents a better fit compared to the Freundlich model isotherm with the experimental data.

**Table 3:** Freundlich isotherm model fitting results

Adsorbents	Intercept	Slope	*K*	*n*	*R* ^2^
g-CN	0.346	0.381	2.217	2.627	0.879
ZCN-1	0.454	0.369	2.846	2.711	0.920
ZCN-2.5	0.395	0.417	2.485	2.398	0.940
ZCN-5	0.349	0.378	2.235	2.648	0.762
ZCN-10	0.244	0.382	1.753	2.621	0.838
ZCN-15	0.109	0.361	1.286	2.768	0.874

The results indicated that the adsorption was a monolayer process and that there were no interactions among the orange II dye molecules. The ZCN composites have a homogeneous character, and no more adsorption occurred at the site of the ZCN composites occupied by the orange II dye molecule.^76^ The maximum adsorption capacity for ZCN-1 and ZCN-2.5 were 15.02 and 15.53 mg g^−1^, respectively, which were slightly higher than that of bare g-CN (13.44 mg g^−1^). The increment adsorption capacity of ZCN-1 and ZCN-2.5 and the decreases adsorption capacity of higher ZnO content composites compared to pure g-CN can be attributed to various factors. The increase in π–π restacking, electron transfer from g-CN to ZnO, and the formation of an internal electric field in the composites enhance the π–π interaction and electrostatic attraction between the g-CN network and the aromatic ring of orange II dye.^19,22^ The probability of hydrogen bond formation between the orange II dye and the g-CN network in the composite may increase due to the reduction of N–H bonds and adsorbed water or moisture content. Furthermore, increasing the total pore volume and average pore size of ZCN-1 and ZCN-2.5 composites can increase the adsorption capacity.^77^ The surface area and crystallinity of these composites did not change significantly compared to pure g-CN. Hence, the adsorption ability of the ZCN-1 and ZCN-2.5 composites was increased compared to pure g-CN. On the contrary, for ZCN composites containing a further increasing amount of ZnO, the surface area and total pore volume are drastically decreased compared to pure g-CN. Average pore diameter values are also decreasing. Furthermore, the crystallinity of these composites was completely diminished. The decrement of factors such as surface area, pore volume, pore diameter, and crystallinity may influence the increment of factors such as the π–π interaction, electrostatic attraction, and hydrogen bond for these composites. Hence, the adsorption ability was decreased compared to pure g-CN.

The maximum monolayer adsorption capacity of orange II dye solution in the present study is compared with different types of adsorbents in the reported literature (Table S2†).^27–33^ It is observed that the prepared composite ZCN-2.5 showed moderate adsorption ability to adsorb orange II dye solution.

The adsorption thermodynamics were investigated by keeping the mixture of orange II dye solution (10 mg L^−1^) and ZCN-2.5 with constant stirring in a dark condition at 20, 30, 40, and 50 °C for 30 minutes. The adsorption of orange II solution onto ZCN-2.5 has been found to decrease with increasing temperature (Fig. S5a†). A number of thermodynamic parameters, including Gibbs free-energy change (Δ*G*^0^), enthalpy change (Δ*H*^0^), and entropy change (Δ*S*^0^) were estimated to inspect the adsorption nature of the present work.^75,78^ The Gibb's free energy change (Δ*G*^0^) is computed by the following eqn:6
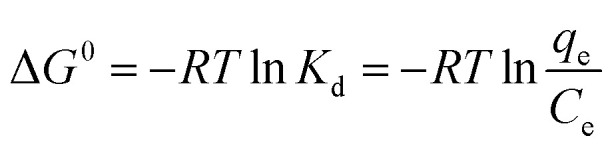
where *R* is the universal gas constant (8.3145 J mol^−1^ K^−1^), *T* is the temperature (K), *K*_d_ is the distribution coefficient (L g^−1^), *q*_e_ is the equilibrium concentration of orange II on the adsorbent ZCN-2.5 (mg g^−1^), and Ce is the equilibrium concentration of orange II in the aqueous phase (mg L^−1^).

The Van't Hoff equation can be used to explain the relationship between enthalpy change and entropy change as follows:7
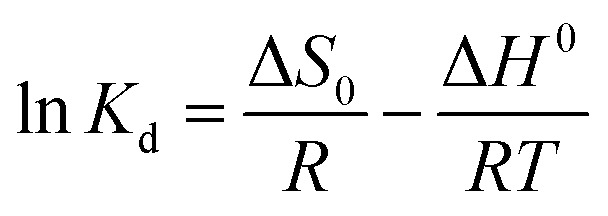


As illustrated in Fig. S5b,† the values of enthalpy change (Δ*H*) and entropy change (Δ*S*) were determined from the slope and intercept of ln *K*_d_*vs.* 1/*T* plots. The values of the estimated thermodynamic parameters are shown in Table 4.

**Table 4:** Thermodynamics parameters for adsorption of orange II dye on ZCN-2.5 composite

Temperature (°C)	Gibbs free energy change (Δ*G*^0^) (kJ mol^−1^)	Enthalpy change (Δ*H*^0^) (kJ mol^−1^)	Entropy change (Δ*S*^0^) (J mol^−1^)
20	0.279	−13.121	−45.568
30	0.638		
40	1.075		
50	1.662		

The positive values of Gibb's free energy change (Δ*G*^0^) suggested that the adsorption of orange II solution onto ZCN-2.5 was not a spontaneous process. Besides, as shown in Fig. S5a† and Table 4, Gibb's free energy change (Δ*G*^0^) increased with increasing temperature, indicating the adsorption decreased with increasing temperature. The negative value of Δ*S* (−45.6 J mol^−1^) exposed the decrease in randomness during the adsorption process. Furthermore, the negative value of Δ*H* (−13.1 KJ mol^−1^) revealed the exothermic nature of the adsorption procedure.

Furthermore, the pH value of the solution affects the surface charge of the adsorbent. Hereafter, the effect of the pH of the solution on the adsorption of the dye can be considered an important factor. The impact of pH on the adsorption of orange II dye solution (10 mg L^−1^) at several pH such as 3, 5.5 (uncontrolled pH), 7, and 10 on ZCN-2.5 was investigated at room temperature (20 °C). As shown in Fig. S6,† the adsorption decreased with the increasing pH of the dye solution. At lower pH, the surface of the composite is positively charged and negatively charged at higher pH. At lower pH, the positively charged surface of the composite may be electrostatically attracted to the anionic orange II dye, which could enhance the adsorption.

### 3.10. Photocatalytic degradation of orange II solution

**Fig. 7:**
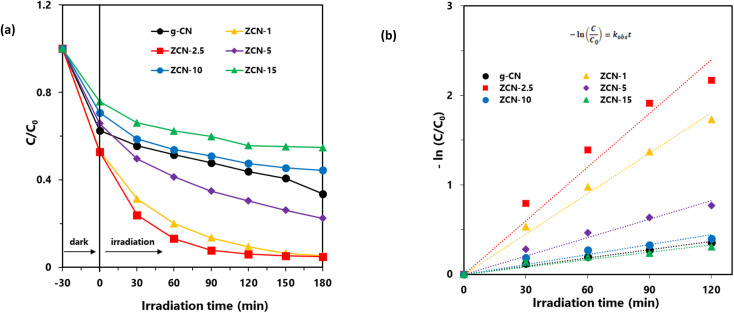
(a) Photocatalytic degradation of orange II dye solution using different photocatalysts with the irradiation of visible light and (b) the plot of −ln(*C*/*C*_0_) *versus* irradiation time; orange II: 10 mg L^−1^ (30 mL), photocatalyst: 30 mg.

The photocatalytic degradation of aqueous orange II dye by using synthesized ZCN composites was investigated under visible light irradiation. As shown in Fig. 7a, the degradation capacity of ZCN composites increased as the amount of ZnO in the composite increased. The ZCN-2.5 composite showed the highest photocatalytic degradation. The increase in visible light absorption ability as well as the decreased optical band gap and higher charge separation of the composites are responsible for enhanced photocatalytic degradation.^79^ The composites containing higher amount of ZnO have very low surface area and crystallinity. Hence, the photocatalytic degradation ability of the composites decreased, further increasing the ZnO content after the ZCN-2.5 composite.

Furthermore, the Langmuir–Hinshelwood (L–H) model was utilized to investigate the reaction rate and kinetics of the photocatalytic degradation process. Eqn (8) represents the L–H model that was developed by Turchi and Ollisand.8
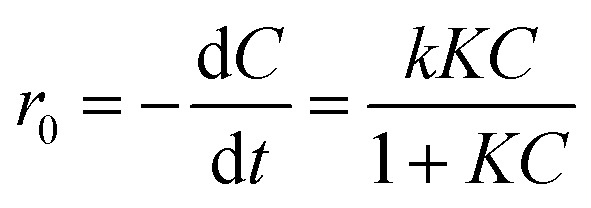
where, *r*_0_ is the rate of the degradation reaction, *k* is the rate constant, and *K* and *C* are the adsorption equilibrium constant and reactant concentration, respectively. If the initial concentration *C*_0_ is very small, it can be shortened to eqn (9) as follows:9
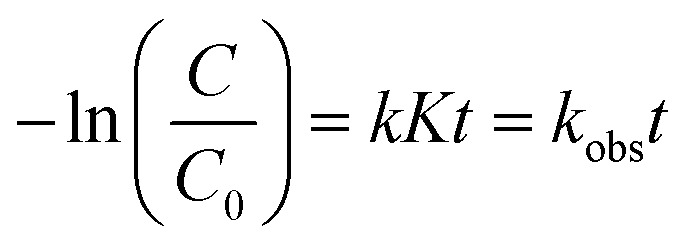
In relation to −ln(*C*/*C*_0_), the equation becomes a linear expression on time *t*, where *k*_obs_ is the reaction rate constant.^80^ So as to check the assumption, −ln(*C*/*C*_0_) was plotted as a function of irradiation time for the degradation of orange II by the photocatalysts. As shown in Fig. 7b, the linear relations were attained as expected. Hence, the degradation process follows pseudo-first-order reaction kinetics. The kinetic parameters, such as rate constant, half-life, and correlation coefficient are presented in Table 5. It was found that the degradation rate of orange II with the ZCN-2.5 composite (0.02 min^−1^) was 6.67 times higher than that obtained with bare g-CN (0.003 min^−1^). Hence, the relative degradation ability of the ZCN-2.5 composite was significantly higher than its relative adsorption ability compared to bare g-CN (Tables 2 and 5).

**Table 5:** Kinetic parameters on photocatalytic degradation of Orange (II) solution (10 mg L^−1^) using pure g-CN and different ZCN composites

Photocatalysts	Rate constant (min^−1^)	Half-life (min)	*R* ^2^
g-CN	0.003	231	0.987
ZCN-1	0.015	46	0.991
ZCN-2.5	0.020	35	0.954
ZCN-5	0.007	100	0.968
ZCN-10	0.004	187	0.889
ZCN-15	0.003	257	0.916

Furthermore, the reusability of the ZCN-2.5 composite was evaluated by five sequential cycles of orange II dye degradation with visible light irradiation. As shown in Fig. S7,† it was observed that the degradation of orange II dye solution using ZCN-2.5 decreased by a negligible amount after the five cycles. These findings suggest that the ZCN-2.5 composite is highly stable and has a repeatable ability to degrade orange II solution under visible light irradiation.

### 3.11. Mechanism

**Fig. 8:**
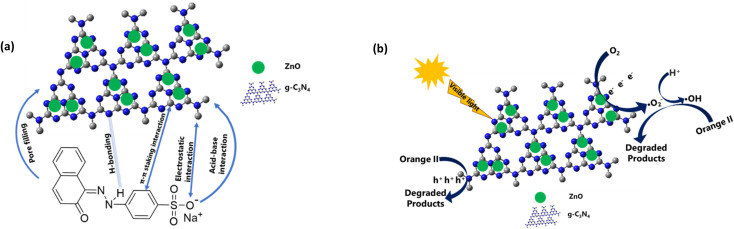
Proposed mechanism of (a) adsorption and (b) photocatalytic degradation of orange II dye solution using ZCN composites.

The removal of orange II dye from aqueous solution using ZCN composites involved adsorption and photodegradation processes. The adsorption is attributed to the π–π interactions, electrostatic interactions, H-bonding, acid–base interactions between composite and dye, and pore filling of the composite by the dye molecule (Fig. 8a). It can be seen that the π conjugations are restacking due to the reforming of the triazine ring of the g-CN network by ZnO.^64^ Furthermore, electrons are transferred from the g-CN network to ZnO and form an internal electric field.^65,66^ These may enhance the π–π interactions with the aromatic benzene ring of orange II dye. The induced positive charge of the g-CN network may attract the negative charge of the dye molecule through electrostatic interactions and enhance adsorption. The heterocyclic N-atoms of the *s*-triazine ring in the ZCN composites and g-CN may interact with the dye molecule through hydrogen bonding, as shown in Fig. 8a.^19^ The g-CN network may have slightly basic properties due to the presence of N atoms.^81^ Hence, acid–base interaction may involve g-CN and the acidic character of orange II during adsorption. Due to the presence of lower quantity of adsorbed water in the composites, the adsorption of orange II on the ZCN composites may increase through available pore filling. On the contrary, due to the excess amount of ZnO in the ZCN composites with higher content of ZnO, the pores of the g-CN framework are blocked, and the surface area is drastically decreased. Hence, adsorption ability decreased.

The photocatalytic degradation reaction involves the interaction of visible light with the ZCN composite and the formation of photocharges (Fig. 8b). Electrons in the valence band of the g-CN were transferred to the conduction band of ZnO through the interfacial charge transfer pathway. As a result, e^−^/h^+^ pair recombination was decreased, and the separation of photocharges was enhanced. Based on the previously reported literature, the reactive species of ˙O_2_^−^, ˙OH, and h^+^ are involved during the photocatalytic degradation reactions.^57,79^ Environmental oxygen was reacted with the photoelectrons of ZnO to create the reactive radical of ˙O^2−^. Then the ˙O^2−^ reacted with H^+^ to form the ˙OH radical. After that, the ˙O_2_^−^ and ˙OH radicals reacted with the orange II dye solution and produced degraded products. The orange II dye was also degraded by the direct reaction of h^+^ in the valence band of g-CN. Due to the extensively lowered surface area and crystallinity, the ZCN composites containing higher ZnO content showed lowered photocatalytic degradation activity.

## 4. Conclusions

In summary, ZCN composites were fabricated by a simple calcination of mixtures of urea and zinc acetate. The adsorption of orange II dye on the ZCN composites is attributed to the π–π interactions, electrostatic interactions, H-bonding, acid–base interactions between the composite and dye, and pore filling of the composites. The adsorption isotherms were fitted with the Langmuir isotherm, and the maximum adsorption ability of ZCN-2.5 was slightly higher than that of bare g-CN. The photocatalytic degradation of orange II dye solution using ZCN composites with visible light irradiation is attributed to the enhanced visible light absorption efficacy and decrement of photogenerated e^−^/h^+^ pair recombination. The photocatalytic degradation followed pseudo-first-order reaction kinetics. The ZCN-2.5 composite showed significantly enhanced photodegradability compared to bare g-CN. Drastically decreased surface area and crystallinity of composites containing higher amount of ZnO are responsible for their lower adsorption and degradation abilities.

## Author contributions

M. H. S.: conceptualization, methodology, data curation, formal analysis, investigation, writing – original draft, and writing – review and editing. A. K.: formal analysis, and writing – review and editing. I. T.: formal analysis, and writing – review and editing. M. F.: formal analysis, and writing – review and editing. H. K.: writing – review and editing. S. K.: writing – review and editing, and supervision.

## Conflicts of interest

There are no conflicts to declare.

## Supplementary Material

RA-014-D4RA01481B-s001
