# Supplementary material for: Purification of aqueous orange II solution through adsorption and visible-light-induced photodegradation using ZnO-modified g-C3N4 composites
Source: RSC Adv. 2024 Jun 4;14(25):17888–900. doi: 10.1039/d4ra01481b (PMC11149495; doi:10.1039/d4ra01481b)
Supplement: RA-014-D4RA01481B-s001 [file RA-014-D4RA01481B-s001.pdf]

Electronic Supplementary Material (ESI) for RSC Advances.
This journal is © The Royal Society of Chemistry 2024

Electronic Supplementary Information

Purification of aqueous orange II solution through adsorption and visible-light-induced photodegradation using ZnO-modified g-C_3N_4 composites

Mahmudul Hassan Suhag,*[a,b] Aklima Khatun,[a] Ikki Tateishi,[c] Mai Furukawa,[a] Hideyuki Katsumata,[a] and Satoshi Kaneco*[a]

[a] Department of Applied Chemistry, Graduate School of Engineering, Mie University, Tsu, Mie 514-8507, Japan

[b] Department of Chemistry, University of Barishal, Barishal 8254, Bangladesh

[c] Mie Global Environment Center for Education & Research, Mie University, Tsu, Mie 514-8507, Japan

* Correspondence: suhag.che057@gmail.com, kaneco@chem.mie-u.ac.jp

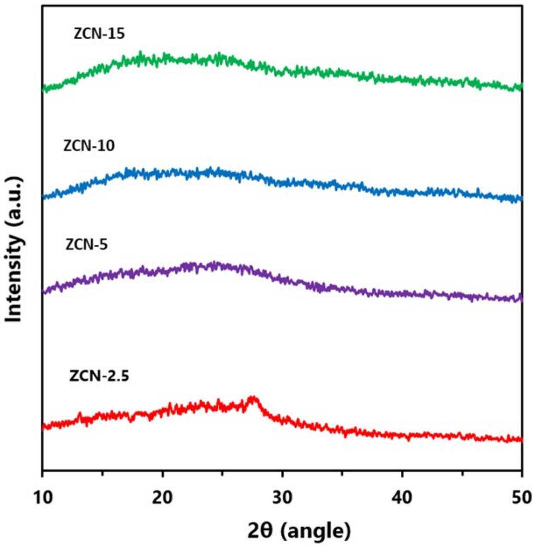

Fig. S1 Enlarged (2θ range 10-50°) XRD patterns of ZCN-2.5, 5, 10 and 15 composites.

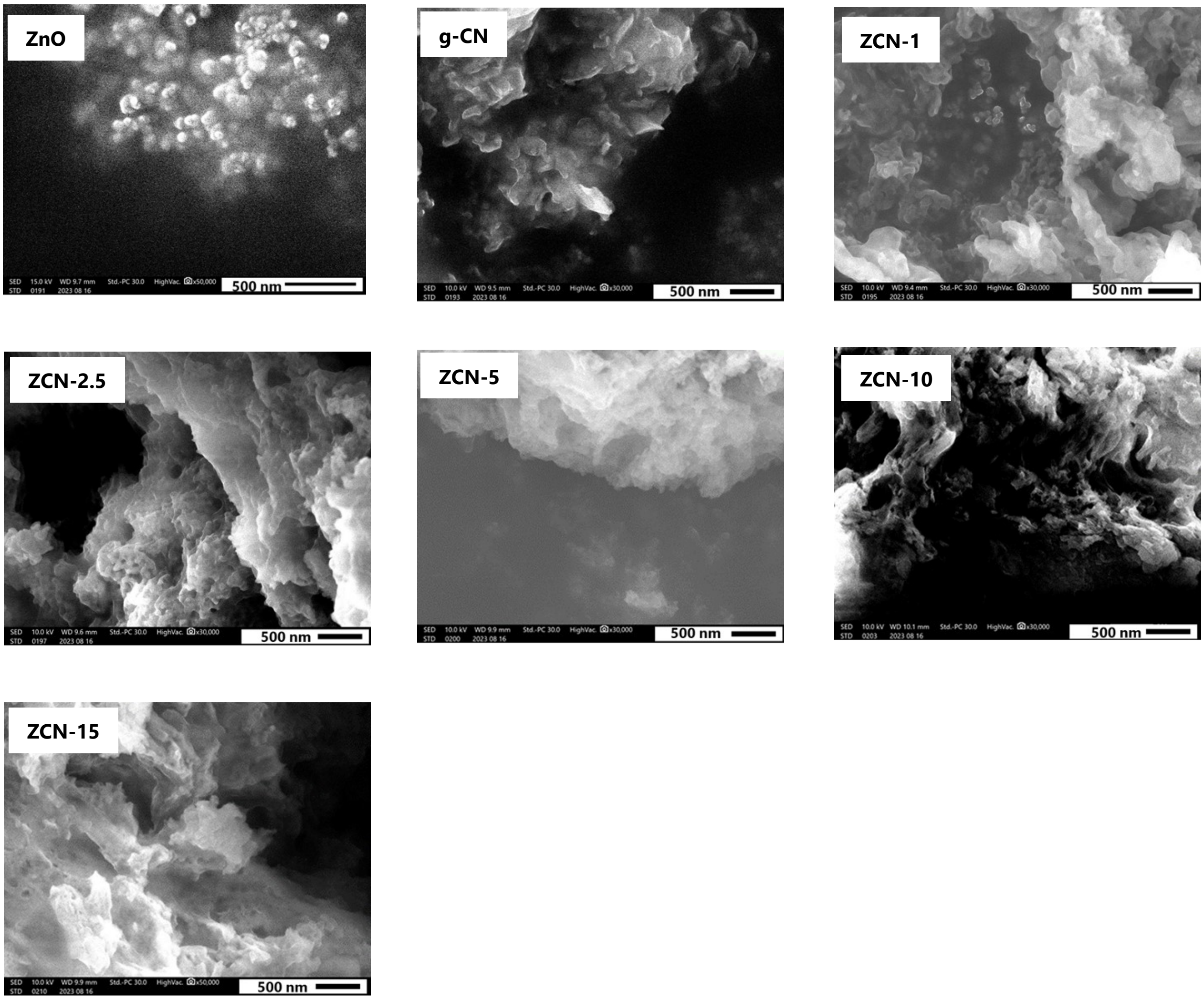

Fig. S2 SEM images of ZCN composites, ZnO, and g-CN.

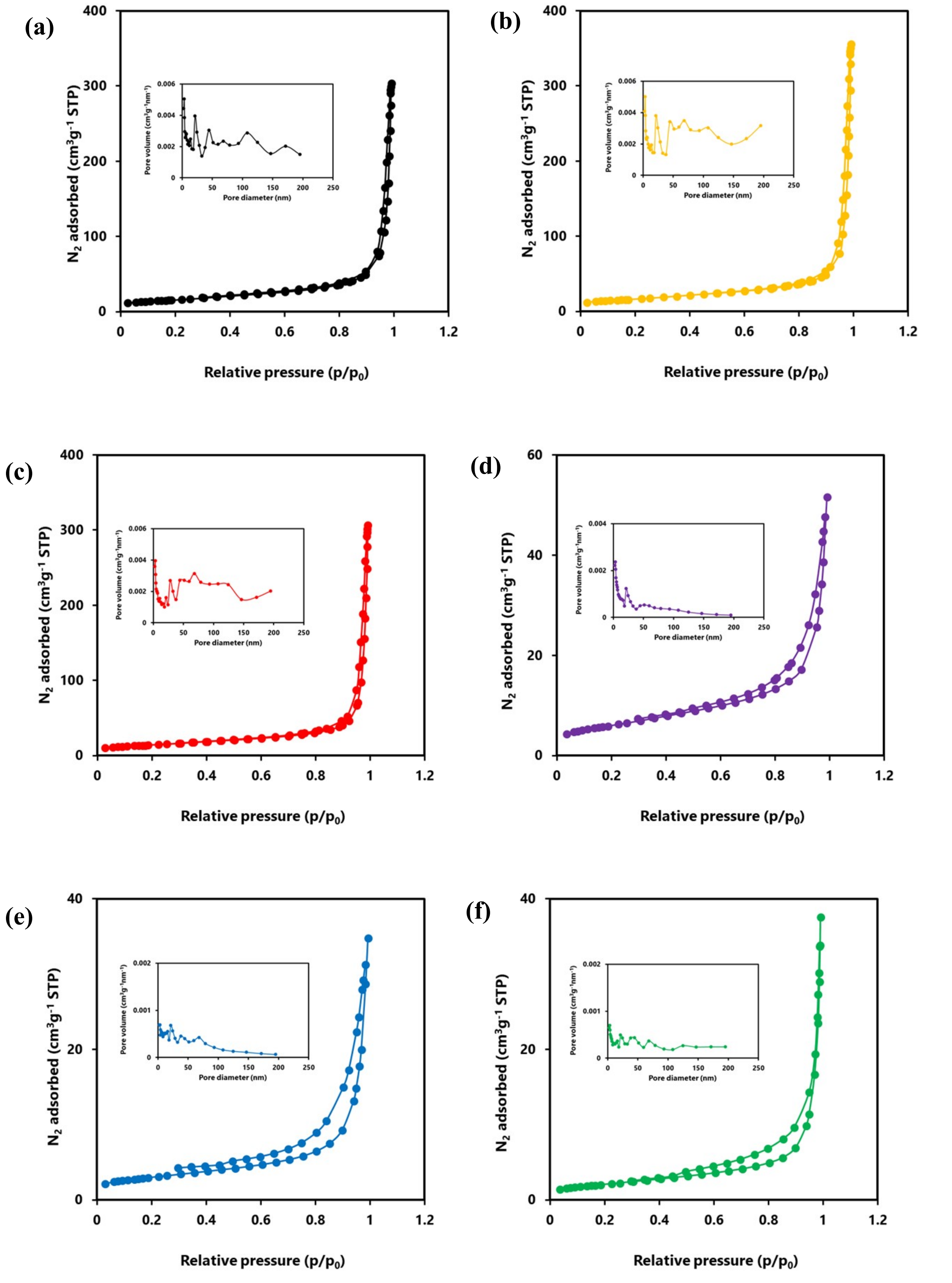

Fig. S3 N_2 adsorption-desorption isotherms (inset: pore size distribution curves) of (a) g-CN, (b) ZCN-1, (c) ZCN-2.5, (d) ZCN-5, (e) ZCN-10, and (f) ZCN-15.

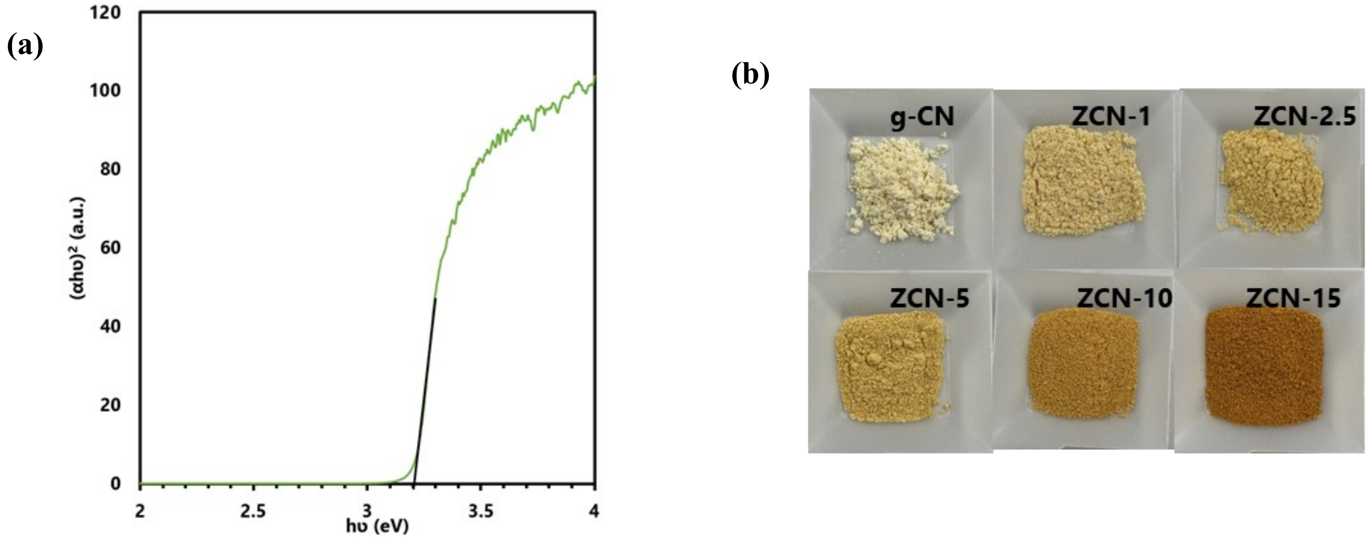

Fig. S4 (a) Tauc plot of ZnO and (b) Photographs for appearance of all ZCN composites and bare g-CN.

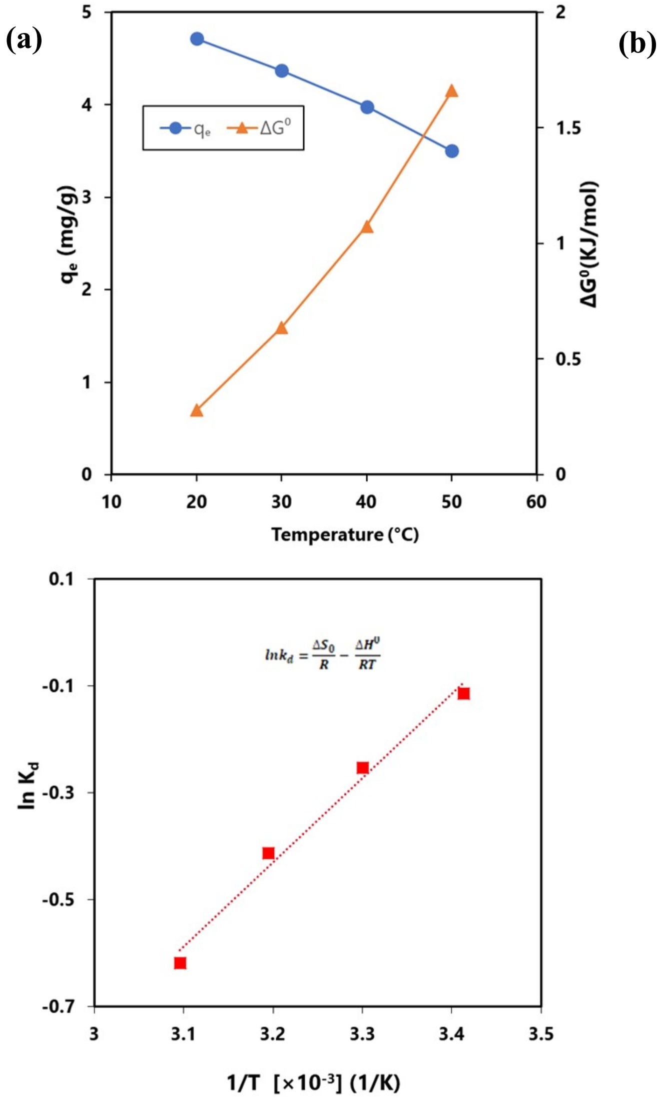

Fig. S5 (a) The relationship between temperature and amount of orange II dye (10 ppm) adsorbed q_e on ZCN-2.5 composite as well as corresponding Gibbs free energy change (ΔG^0); (b) the plot of In K_d versus 1/T.

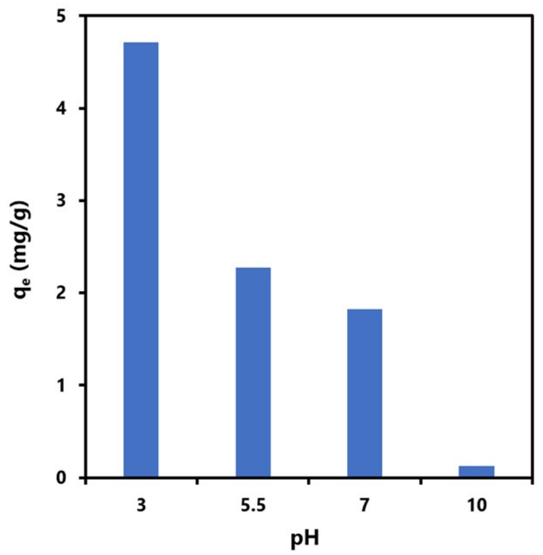

Fig. S6 The effect of pH for adsorption of orange II dye (10 ppm) on ZCN-2.5 composite.

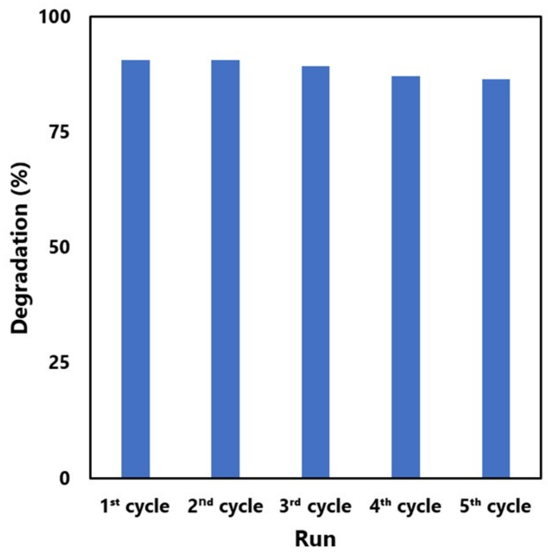

Fig. S7 Photocatalytic reusability of ZCN-2.5 composite on photocatalytic degradation of orange II solution.

Table S1 Surface atomic ratios of g-CN and ZCN-2.5 composite

Samples	C (%)	N (%)	O (%)	Zn (%)	C/N molar ratio	C/O molar ratio
g-CN	43.8	56.0	0.1	-	0.78	438
ZCN-2.5	43.2	51.0	3.5	2.2	0.85	12.34

Table S2 Comparison of the maximum monolayer adsorption capacities of Orange II onto synthesized ZCN-2.5 and various reported adsorbents

Adsorbents	Maximum monolayer adsorption capacity, q_{max} (mg g^{-1})	References
Biosolid adsorbent	0.60	27
Spent brewery grains	30.47	28
Activated carbon cloths	90.00	29
Rice husk ash	59.10	30
Rice husk/$CoFe_2O_4$ composite	65.00	30
Rice husk/$MnFe_2O_4$ composite	69.40	30
Bottom ash	12.51	31
De-oiled soya	8.88	31
Chitosan	116.00	32
Unmodified zeolite	0.63	33
Surfactant-modified zeolite	3.62	33
ZCN-2.5	15.53	Present study
